# Prognostic Role of Immune Checkpoint Regulators in Cholangiocarcinoma: A Pilot Study

**DOI:** 10.3390/jcm10102191

**Published:** 2021-05-19

**Authors:** Lu Cao, Prashanth Prithviraj, Ritu Shrestha, Revati Sharma, Matthew Anaka, Kim R. Bridle, George Kannourakis, Darrell H.G. Crawford, Aparna Jayachandran

**Affiliations:** 1 Gallipoli Medical Research Institute, Greenslopes Private Hospital, Brisbane, QLD 4120, Australia; l.cao1@uq.edu.au (L.C.); ritu.shrestha@uq.edu.au (R.S.); k.bridle@uq.edu.au (K.R.B.); d.crawford@uq.edu.au (D.H.C.); 2 Faculty of Medicine, University of Queensland, Brisbane, QLD 4120, Australia; 3 Fiona Elsey Cancer Research Institute, Ballarat, VIC 3350, Australia; Prashanth@ballaratoncology.com.au (P.P.); revati@fecri.org.au (R.S.); george@fecri.org.au (G.K.); 4 School of Science, Psychology and Sports, Federation University Australia, Ballarat, VIC 3350, Australia; 5 Department of Medical Oncology, University of Alberta, Edmonton, AB T6G 1Z2, Canada; matt.anaka@gmail.com

**Keywords:** immune checkpoints, EMT, PD-L1, cancer stem cells, cholangiocarcinoma, CD73, Galectin-9, IDO1

## Abstract

Cholangiocarcinoma (CCA) is a hepatobiliary malignancy associated with steadily increasing incidence and poor prognosis. Ongoing clinical trials are assessing the effectiveness and safety of a few immune checkpoint inhibitors (ICIs) in CCA patients. However, these ICI treatments as monotherapies may be effective for a proportion of patients with CCA. The prevalence and distribution of other immune checkpoints (ICs) in CCA remain unclear. In this pilot study, we screened databases of CCA patients for the expression of 19 ICs and assessed the prognostic significance of these ICs in CCA patients. Notably, expression of immune modulator IDO1 and PD-L1 were linked with poor overall survival, while FASLG and NT5E were related to both worse overall survival and progression-free survival. We also identified immune modulators IDO1, FASLG, CD80, HAVCR2, NT5E, CTLA-4, LGALS9, VTCN1 and TNFRSF14 that synergized with PD-L1 and correlated with worse patient outcomes. In vitro studies revealed that the expression of ICs was closely linked with aggressive CCA subpopulations, such as cancer stem cells and cells undergoing TGF-β and TNF-α-mediated epithelial-to-mesenchymal transition. These findings suggest that the aforementioned IC molecules may serve as potential prognostic biomarkers and drug targets in CCA patients, leading to lasting and durable treatment outcomes.

## 1. Introduction

Cholangiocarcinoma (CCA) is the second most common primary liver malignancy. Cholangiocytes, the epithelial cells that line the biliary tree, undergo neoplastic transformation resulting in the formation of CCA [1]. CCA can be categorized as intrahepatic, perihilar, or distal subtypes and accounts for 10–20% of all hepatobiliary malignancies [2]. While potentially curable with surgery if diagnosed at an early stage, the overall clinical outcome of CCA continues to be poor due to its propensity for early local invasion, distant metastasis and high recurrence [3,4]. Furthermore, the majority of patients with CCA are diagnosed at advanced stages, and the administration of chemotherapy has shown limited efficacy [5]. Given the aggressive disease course and lack of targeted treatment options for CCA patients, there is a pressing need for new and efficacious treatment modalities for this malignancy.

Immunotherapy, especially immune checkpoint inhibitors (ICIs), has proven to be an effective therapeutic modality in several chemoresistant malignant diseases including melanoma, renal and non-small cell lung cancers [6]. Immune checkpoints (ICs) maintain self-tolerance and, during an immune response, ICs protect normal tissue from damage [7]. However, tumor cells frequently exploit these ICs, serving as a prominent tumor immune evasion mechanism causing T-cell inactivation and downregulation of T-cell responses [7,8]. Immune checkpoint blockade strategies have been effective in reanimating the T-cell antigen-specific response and associated antitumor effects [8]. Within the tumor microenvironment, ICs may serve as therapeutic targets for the treatment of primary liver malignancies including hepatocellular carcinoma (HCC) and CCA. ICIs hold great promise for HCC and CCA as the deregulation of the immune system contributes to the pathogenesis of these liver malignancies [9,10].

In the past few years, ICIs as monotherapy have elicited a durable and robust antitumor response in only a proportion of cancer patients, with variability both between types of cancers and between patients who share a histological type [11,12]. A trial of monotherapy with anti-PD1 antibody pembrolizumab in phase I/II in CCA patients showed a low response rate (10% to 20%), and little is known of the underlying mechanism of resistance [11,13]. Another phase I trial for CCA patients revealed that the combined treatment with anti-PD-1 antibody nivolumab and cisplatin plus gemcitabine was more effective than nivolumab as monotherapy [14]. Similarly, combining anti-PD-L1 antibody durvalumab with the anti-CTLA4 antibody tremelimumab was more effective than monotherapies in CCA patients [15].

Analysis of immune checkpoint marker testing such as PD-L1 alone for patient selection and predicting response to ICI therapy has proven insufficient in many cancers [16]. Therefore, identifying and characterizing additional predictive biomarkers are of the utmost importance for the selection of a subset of CCA patients who are more likely to respond to ICI therapies. A better understanding of alternative checkpoint pathways may be required to increase the clinical benefits of ICIs in CCA patients. These alternative checkpoints may provide additional targets for rational combinatorial therapies that may enhance the effects of immunotherapy in CCA.

Combining the expression of immune checkpoints with additional biomarkers such as those identifying epithelial-to-mesenchymal transition (EMT) and cancer stem cells (CSCs) are also being considered in the management of some cancers [10,17,18]. EMT is defined by the loss of epithelial properties and the concomitant gain of mesenchymal properties. EMT contributes to the invasion and metastasis of tumor cells and also to immunosuppression [19,20]. A correlation between the EMT phenotype with multiple ICs in many patient tumors has been reported [10,21]. In extrahepatic CCA, a close relationship between EMT and PD-L1 expression has been reported [22]. However, little is known regarding the association of other ICs with an EMT phenotype in CCA. CSCs are also referred to as tumor-initiating or tumor-propagating cells. CSCs represent a small subpopulation of cells within the tumor that contributes to tumor initiation, metastasis and recurrence. CSCs are endowed with self-renewal, pluripotent properties and enhanced resistance to chemotherapy compared to the tumor bulk [23]. The ability of PD-L1 to inhibit cancer stemness in CCA has been demonstrated [24]. Other studies have reported the expression of PD-L1 and PD-L2 in CSCs derived from colon and breast cancers [25]. Little is known regarding the association between expressions of other immune modulators and CCA-related CSC phenotype.

In this pilot study, we sought to identify prognostic immune-modulatory molecules in CCA patients. To this end, we analyzed CCA patient databases from The Cancer Genome Atlas (TCGA) and SurvExpress [26,27]. We correlated the expression of immune-related molecules with patient prognosis. Given that EMT and CSCs have a substantial role in CCA initiation and progression, we assessed the association of immune checkpoint molecule expression in aggressive CCA cell subpopulations such as CSCs and cells undergoing transforming growth factor (TGF)-β1- and tumor necrosis factor (TNF)-α-mediated EMT.

## 2. Materials and Methods

cBioPortal OncoPrint evaluation of immune checkpoint molecules: cBioPortal OncoPrint (http://cbioportal.org, accessed on 10 May 2021) was used to generate a graphical summary of gene expression changes in immune checkpoint molecules across CCA patient samples. Within cBioPortal, we utilized the Cholangiocarcinoma (TCGA, Firehose Legacy) case set of 51 patients to evaluate gene changes in immune checkpoint genes. CCA patients are represented as columns and immune-modulatory genes are represented as rows. Genomic alterations, including copy number aberrations, changes in gene or protein expressions and mutations, are represented by glyphs and color codes [27].

CCA patient databases: SurvExpress utilizes a gene expression database of different cancers to generate survival analyses of CCA patients (http://bioinformatica.mty.itesm.mx:8080/Biomatec/SurvivaX.jsp, accessed on 10 May 2021). SurvExpress provided a CCA database of 35 patient samples (CHOL-TCGA Cholangiocarcinoma).

Evaluation of immune checkpoint molecules as prognostic biomarkers in CCA patients: SurvExpres was applied to evaluate the relationship between the expressions of 19 immune modulators with the survival of CCA patients based on a Cox regression analysis. The overall survival for CCA patients was estimated by Kaplan–Meier curves. The average intensity of quantile-normalized array data was used for genes with multiple probe sets. Survival and progression-free survival analyses were also performed using a CCA dataset of 36 patients in cBioPortal.

Cell culture and reagents: Prof. Mark Gorrell, Centenary Institute, Australia kindly gifted human CCA cell lines HuCCT-1 and CCLP-1. Human CCA cell line EGI-1 was sourced from Prof. John Mariadason, Olivia Newton-John Cancer Research Institute, Heidelberg, VIC, Australia. MycoAlert tests (ABM, Richmond, BC, Canada) confirmed the mycoplasma-free status of these cell lines. HuCCT-1 was cultured in Roswell Park Memorial Institute (RPMI); 1640 medium (Thermo Fisher Scientific Australia, Scoresby, VIC, Australia) supplemented with 10% fetal bovine serum (FBS) (Gibco, Life Technologies Australia Pty Ltd, Mulgrave, VIC, Australia) and 0.05% Gentamicin (Thermo Fisher Scientific Australia, Scoresby, VIC, Australia). Dulbecco’s modified Eagle’s medium (DMEM) (Thermo Fisher Scientific Australia, Scoresby, VIC, Australia) supplemented with 10% FBS (Gibco, Life Technologies Australia Pty Ltd, Mulgrave, VIC, Australia) and 0.05% Gentamicin (Thermo Fisher Scientific Australia, Scoresby, VIC, Australia) was used to culture CCLP-1. EGI-1 was cultured in DMEM (Thermo Fisher Scientific Australia, Scoresby, VIC, Australia) with 10% FBS (Gibco, Life Technologies Australia Pty Ltd, Mulgrave, VIC, Australia) and 1% penicillin/streptomycin (P/S) (Thermo Fisher Scientific Australia, Scoresby, VIC, Australia). Cells were cultured under a humidified atmosphere with 5% CO_2_ in the air at 37 °C. The cytokines TGF-*β*1 and TNF-α were procured from PeproTech, Cranbury, NJ, USA.

3-dimensional sphere enrichment assay: Trypsin-EDTA was used to detach cells grown as monolayers. Cells were suspended in serum-free stem cell medium following the removal of serum with 1 × PBS washes [28]. The serum-free stem cell medium was prepared with DMEM/F12 medium (Thermo Fisher Scientific Australia, Scoresby, VIC, Australia) supplemented with 20 ng/mL recombinant human epidermal growth factor (rhEGF) (PeproTech, Cranbury, NJ, USA), 10 ng/mL recombinant human fibroblast growth factor (rhFGF) (PeproTech, Cranbury, NJ, USA), 2% B27 supplement without vitamin A (Invitrogen, Scoresby, VIC, Australia) and 1% N2 supplement (Invitrogen, Scoresby, VIC, Australia). Then, 5000 cells were plated per well in ultra-low attachment, 6-well plates (Corning, Melbourne, VIC, Australia). Cells were cultured in a humidified atmosphere of 5% CO_2_ in air at 37 °C for 7 days. The spheres were collected by gentle centrifugation.

RNA extraction and cDNA synthesis: ISOLATE II RNA Mini Kit (Bioline, Eveleigh, NSW, Australia) was used for the purification of RNA [29]. A NanoDrop 2000c spectrophotometer (Thermo Fisher Scientific Australia, Scoresby, VIC, Australia) was used to confirm RNA quantity and purity. Then, 1 µg RNA was reverse transcribed to cDNA with a Bioline SensiFAST cDNA Synthesis Kit (Bioline, Eveleigh, NSW, Australia).

Quantitative reverse transcription-PCR (qRT-PCR): Applied Biosystems ViiA 7 Real-Time PCR System was used for performing qRT-PCR with Lo-ROX SYBR Green (Bioline, Eveleigh, NSW, Australia) [28]. Briefly, a 3-step cycle of the following conditions, 95 °C for 5 s, 63 °C for 20 s and 75 °C for 20 s was repeated for 40 cycles. *Beta-Actin* (*ActB*) was used as the housekeeping gene. Table 1 lists the primers used in this study. The 2ΔΔCt method was used for data analysis. In this 2ΔΔCt method, candidate gene expression was normalized to *ActB* expression, and copies of target gene per 10,000 copies of *ActB* was used to present data.

Western blot analysis: Western blot analyses were performed as previously described [29]. Briefly, cells were cultured and treated in 6-well plates. RIPA buffer (Thermo Fisher Scientific Australia, Scoresby, VIC, Australia) with Complete (Roche, Australia) and PhosSTOP (Roche, Sydney, NSW, Australia) protease and phosphatase inhibitors were used to lyse cells at 4 °C. Pierce BCA Protein Assay Kit (Thermo Fisher Scientific Australia, Scoresby, VIC, Australia) was utilized to measure total protein concentration. Then, 10 µg of protein was separated by electrophoresis (SDS-PAGE) in a polyacrylamide gel containing sodium dodecyl sulphate (SDS) and transferred to a polyvinylidene difluoride film (PVDF) membrane. Additionally, 5% skim milk in Tris-buffered saline containing 0.1% Tween 20 (TBS-T) was used to block the membranes. Next, the membranes were exposed to primary antibodies at 4 °C overnight. SuperSignal West Femto Maximum Sensitivity Substrate (Thermo Fisher Scientific Australia, Scoresby, VIC, Australia) detected the proteins on the membranes after exposure to HRP-conjugated secondary antibodies. β-Actin was the housekeeping control. Image Quant LAS 500 was used for image capture. Image Studio™ Lite v5.2 software was used for quantification. Table 2 lists the antibodies used in this study.

Statistical analysis: Kaplan–Meier analysis was used to determine the relationship between immune modulator or EMT or CSC expression and CCA patient survival. A log-rank test was performed, and the *p*-value for survival analysis was generated [30]. In vitro experiments were repeated at least thrice, and representative results are presented. Using the Kolmogorov–Smirnov Test of Normality, we determined the normal distribution of the in vitro data sets (data not shown). Comparisons of in vitro data were performed with Student’s two-tailed *t*-test with GraphPad Prism software version 8.00 (GraphPad Software Inc., San Diego, CA, USA). Statistical significance was set at * *p* < 0.05, ** *p* < 0.01, *** *p* < 0.005 and **** *p* < 0.001.

## 3. Results

### 3.1. The Expression of Immune Checkpoint Genes in CCA

To identify ICs involved in CCA immune escape, we examined a panel of 19 immune checkpoint genes previously shown to be associated with patient prognosis in other malignancies. These included both immune-stimulatory and inhibitory genes, namely: *FASLG*, *Galectin-9* (*LGALS9*), *LAG-3*, *TIM-3 (HAVCR2)*, *VSIR*, *VTCN1 (B7-H4)*, *IDO-1*, *TNFRSF9 (CD137)*, *TNFRSF14 (HVEM)*, *TIGIT*, *CD276 (B7-H3)*, *CD27*, *PD-L1 (CD274)*, *PD-L2 (PDCD1LG2)*, *NT5E (CD73)*, *CD80*, *TNFRSF18 (GITR)*, *BTLA* and *CD28*. OncoPrint analysis in cBioPortal was performed to evaluate the expression and any possible genetic changes associated with these ICs in the tumors of CCA patients (*n* = 51). This patient cohort included 35 patients with intrahepatic cholangiocarcinoma, 9 patients with distal cholangiocarcinoma and 7 patients with perihilar cholangiocarcinoma. Clinical data of the patient cohort is listed in Table 3.

**Figure 1:**
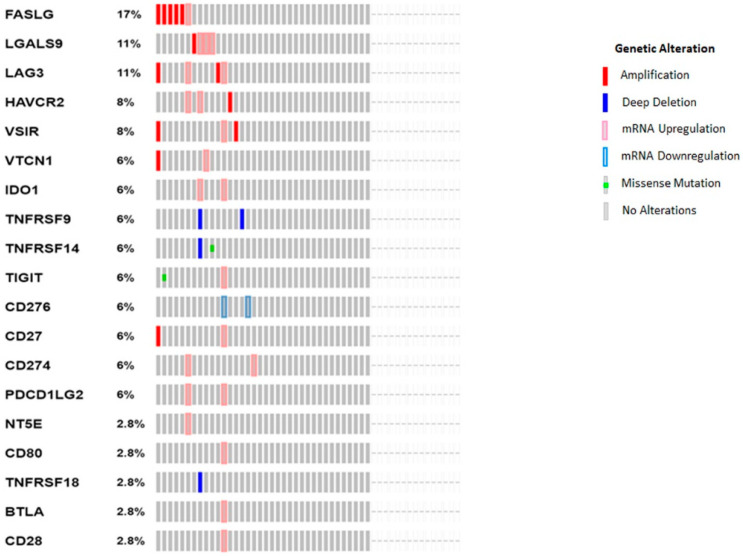
The OncoPrint analyses of CCA patients reveal changes in expression of ICs. Rows and columns represent genes and CCA patients, respectively. Genetic alterations, such as copy number alterations (homozygous deletions and amplifications), mutations and gene expression changes, are represented by glyphs and color codes. The patient order is presented as per alterations.

The OncoPrint analyses revealed FASLG (17%) amplification and mRNA upregulation, *LGALS9* (11%), *LAG3* (11%), *HAVCR2* (8%), *VSIR* (8%), *VTCN1* (6%) and *CD27* (6%) (Figure 1). mRNA upregulation was identified in *IDO1* (6%), *CD274* (6%), *PDCD1LG2* (6%), *NT5E* (2.8%), *CD80* (2.8%), *BTLA* (2.8%) and *CD28* (2.8%). Briefly, mRNA downregulation was seen for *CD276* (6%). Deep deletion was noted in *TNFRSF9* (6%) and *TNFRSF18* (2.8%). Deep deletion and missense mutation were identified in *TNFRSF14* (6%), while missense mutation and mRNA upregulation was noted in *TIGIT* (6%) (Figure 1). CCA may warrant immunotherapeutic approaches due to increased distribution of immune checkpoint molecules in CCA patients.

### 3.2. Immune Biomarkers Prognosticate Poor Survival in CCA Patients

**Figure 2:**
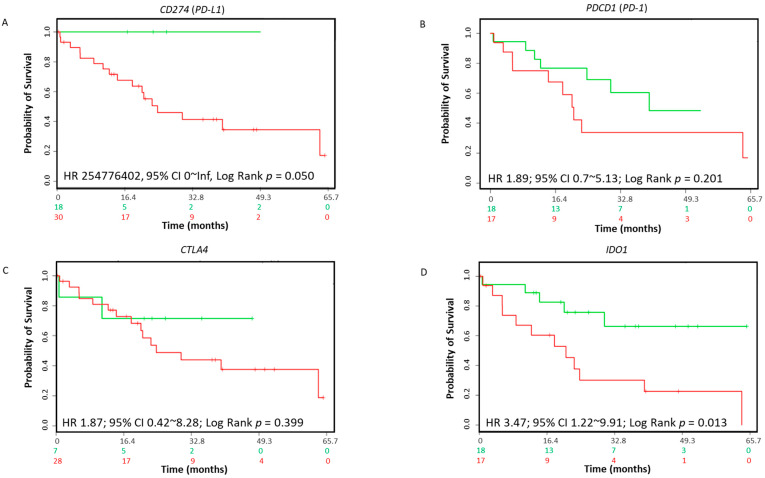
Association between immune modulators and survival in CCA patients. The Kaplan–Meier survival curves in SurvExpress CCA patients for the gene expression of (**A**) *CD274 (PD-L1)*, (**B**) *PDCD1* (*PD-1)*, (**C**) *CTLA4* and (**D**) *IDO1*. Low-risk groups are indicated in green and high-risk groups are indicated in red. The x-axis presents the study time in months. HR, CI and *p*-values are shown in the insert.

To investigate the prognostic value of ICs in CCA, we evaluated the SurvExpress CCA dataset to evaluate the overall survival in CCA patients. In this cohort of CCA patients, we evaluated whether the expression of putative ICs, *PD-L1* (*CD274*), *PD-1* (*PDCD1*) and *CTLA4* were associated with overall survival. No correlation of these ICs with overall survival was observed in CCA patients (Figure 2A–C). Notably, the altered expression of *IDO1* (hazard ratio (HR): 3.47; 95% confidence interval (CI): 1.22~9.91; log-rank equal curves *p* = 0.013) in the 35-CCA-patient cohort was linked with overall worse survival (Figure 2D). Similarly, other immune checkpoint gene expressions showed no correlation with overall CCA patient survival (Table 4). In cBioPortal, the TCGA CCA 36-patient cohort, *FASLG* (log-rank test *p* = 1.370 × 10^−3^) and *NT5E* (log-rank test *p* = 2.826 × 10^−3^) expressions correlated with lower overall survival (Table 5). Similarly, *FASLG* (log-rank test *p* = 3.843 × 10^−3^) and *NT5E* (log-rank test *p* = 4.072 × 10^−3^) expression correlated with lower progression-free survival in the 36 CCA patient cohort (Table 5).

### 3.3. Association of Clinicopathological Characteristics with Expression of IDO1, FASLG and NT5E in CCA Patients

We next interrogated whether ICs significantly associated with poor prognosis can function as independent risk factors for prognosis prediction in CCA patients. Table 6 shows the relationship between pathological features and expression of *IDO1*, *FASLG* and *NT5E* in CCA patients in the cBioPortal database. Chi-squared tests were used to compare the clinicopathological data. *IDO1* expression in CCA patients was associated with neoplasm disease stages II and III (*p* = 1.874 × 10^−3^). Patients with altered *IDO1* expression was associated with a presence of a risk factors for HCC, including smoking and hepatitis B (*p* = 0.012).

A positive association between *FASLG* expression was associated with the neoplasm disease lymph node stage in CCA patients (*p* = 0.041), where 20% of patients with altered *FASLG* expression were in the American Joint Committee on Cancer (AJCC) lymph node stage N1. Moreover, 60% of CCA patients with *FASLG* expression were in the AJCC metastasis stage M0 exhibiting no metastasis (*p* = 0.018). However, 40% of patients with altered *FASLG* expression were in the MX stage with no information available on metastasis status. All patients with *NT5E* expression were in MX and NX of AJCC metastasis and lymph node stages, respectively, where no information was available on metastasis or lymph node status.

*IDO1*- (*p* = 0.049), *FASLG*- (*p* = 1.046e-3) and *NT5E*-expressing (*p* = 3.565e-3) CCA patients were not administered neoadjuvant therapy prior to resection. Expression of these genes was not associated with clinical parameters such as vascular invasion, gender and AJCC tumor stage code T1–T3.

### 3.4. Coordinate Expression of PD-L1 (CD274), PD-1 and CTLA-4 and Immune Checkpoint Genes in CCA Patients

**Figure 3:**
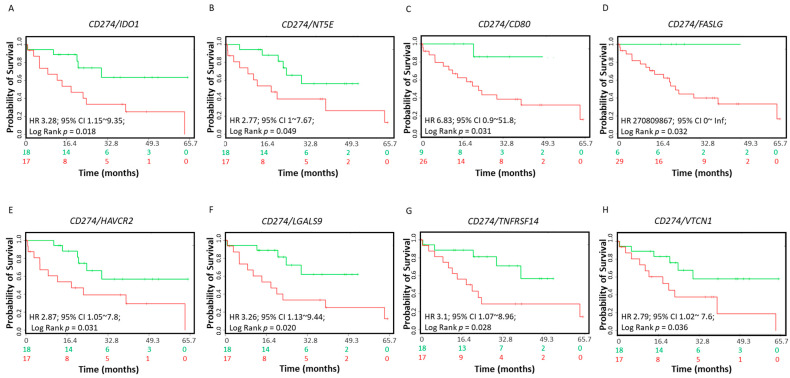
Relationship of immune modulators in combination with *CD274* (*PD-L1*) and survival in CCA patients. The Kaplan–Meier survival curves in SurvExpress CCA patients for the gene expression of (**A**) *CD274*/*IDO1*, (**B**) *CD274*/*NT5E*, (**C**) *CD274*/*CD80*, (**D**) *CD274*/*FASLG*, (**E**) *CD274*/*HAVCR2*, (**F**) *CD274*/*LGALS9*, (**G**) *CD274*/*TNFRSF14* and (**H**) *CD274*/*VTCN1*. Low-risk groups are indicated in green and high-risk groups are indicated in red. The x-axis presents the study time in months. HR, CI and *p*-values are shown in the insert.

Varying degrees of clinical responses to ICI treatments, including anti-PD-L1, anti-PD-1 or anti-CTLA-4, have been noted in different tumor types. Research has focused on identifying predictive biomarkers to stratify patients to increase the rate of response to ICI therapies. In CCA, the coordinated expression of other ICs with PD-L1, PD-1 and CTLA-4 have not been previously explored. We evaluated whether the coordinate expression of ICs with *PD-L1*, *PD-1* and *CTLA-4* showed survival benefits. Coordinate expression of *IDO1* (HR: 3.28, CI: 1.15~9.35, log-rank equal curves *p* = 0.018), *CD73* (HR: 2.77, CI: 1~7.57, log-rank equal curves *p* = 0.040), *CD80* (HR: 6.83, CI: 0.9~51.8, log-rank equal curves *p* = 0.031), *FASLG* (HR: 270809867, CI: 0~inflog-rank equal curves *p* = 0.032), *HAVCR2* (HR: 2.87, CI: 1.05~7.8, log-rank equal curves *p* = 0.039), *LGALS9* (HR: 3.25, CI: 1.13~9.44, log-rank equal curves *p* = 0.020), *TNFRSF14* (HR: 3.1, CI: 1.07~8.96, log-rank equal curves *p* = 0.037) and *VTCN1* (HR: 2.79, CI: 1.02~7.6, log-rank equal curves *p* = 0.036) showed significantly worse overall survival when combined with *PD-L1* (Figure 3A–H). The other 10 ICIs in combination with *PD-L1* did not show a significant association with survival in CCA patients (Supplementary Materials Table S1).

Combining the expression of *TIGIT* (HR: 3.43, CI: 1.15~9.96, log-rank equal curves *p* = 0.016), *TNFRSF14* (HR: 3.06, CI: 1.06~8.84, log-rank equal curves *p* = 0.029), *CTLA4* (HR: 3.02, CI: 1.08~8.45, log-rank equal curves *p* = 0.027) and *FASLG* (HR: 2.99, CI: 1.06~8.4, log-rank equal curves *p* = 0.003) with *PD-1 (PDCD1)* showed worse overall survival (Supplementary Materials Figure S1A–D). *IDO1* (HR: 3.99, CI: 1.39~11.44, log-rank equal curves *p* = 0.005) and *PD-L1* (HR: 270809867, CI: 0~inf, log-rank equal curves *p* = 0.032) revealed worse overall survival in combination with *CTLA-4* (Supplementary Materials Figure S1E,F).

### 3.5. Anchorage-Independent Three-Dimensional CCA Spheres Express Embryonic Stemness and CSC Markers

**Figure 4:**
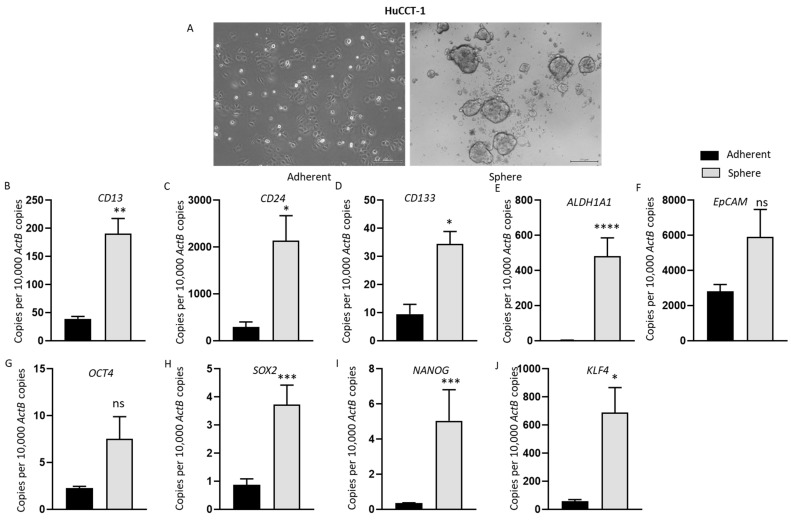
Anchorage-independent three-dimensional spheroid culture enriches HuCCT-1 CSCs. (**A**) Monolayer culture and 3-D culture of HuCCT-1 cells (scale bar = 200 µm). (**B**–**J**) qRT-PCR analysis demonstrated increased expression of embryonic stemness and surface CSC markers in HuCCT-1 spheres compared with HuCCT-1 adherent monolayer culture. Values are mean ± SD of three experiments in triplicate (* *p* < 0.05, ** *p* < 0.01, *** *p* < 0.005, **** *p* < 0.001, ns: not significant). *ActB*: *β-Actin*.

**Figure 5:**
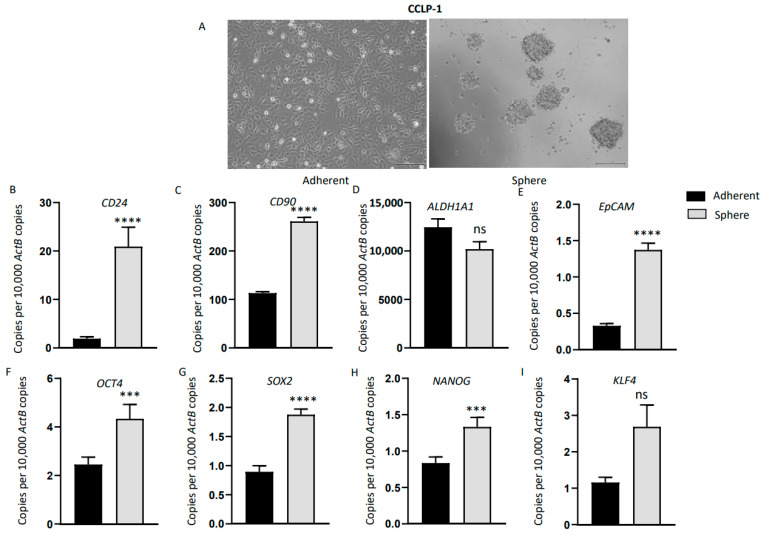
Anchorage-independent three-dimensional spheroid culture of CCLP-1 cells enriches CSCs. (**A**) Monolayer culture and 3-D culture of CCLP-1 cells (scale bar = 200 µm). (**B**–**I**) Enhanced expression of embryonic stemness and cell surface CSC markers were observed in CCLP-1 spheres compared with CCLP-1 adherent monolayer culture by qRT-PCR. Values are mean ± SD of three experiments in triplicate (*** *p* < 0.005, **** *p* < 0.001, ns: not significant). *ActB: β-Actin*.

To assess the relationship between immune checkpoint modulators and CSCs, three-dimensional spheres were generated using human CCA cell lines HuCCT-1, CCLP-1 and EGI-1. By day 7, anchorage-independent spheres were enriched in all CCA cell lines cultured in serum-free stem cell medium (Figure 4A, Figure 5A and Figure S2A). We confirmed the stemness of CCA-derived spheres by examining the expression of stemness genes essential for the proliferation, self-renewal and differentiation of stem cells, including *CD13*, *CD24*, *CD44*, *CD90*, *CD133*, *ALDH1A1*, epithelial cell adhesion molecule (*EpCAM*), Krüppel-like factor 4 (Klf4) and octamer-binding transcription factor 4 (*OCT4*). These CSC markers have been widely used individually or in combination to characterize CSCs in CCA [18,31,32]. As controls, CCA cell lines were grown as adherent monolayers at the same density as the spheres. Three-dimensional sphere cultures and adherent cultures were subjected to RNA extraction on day seven. HuCCT-1 spheres showed markedly elevated expression of stemness markers *CD13*, *CD24*, *CD133*, *ALDH1A1* and *EpCAM* compared with parental adherent cells (Figure 4B–F). In comparison with parental adherent cells, HuCCT-1 spheres also showed upregulation in the expression of the embryonic stem-cell-associated genes *OCT4*, *Sox2*, *Nanog* and *Klf4* (Figure 4G–J). Likewise, CCLP-1-derived spheres showed increased expression of stemness markers *CD24*, *CD90* and *EpCAM* compared with adherent parental cells (Figure 5B–E). In comparison with the adherent parental cells, CCLP-1 spheres showed higher mRNA levels of *Oct4*, *Sox2*, *Nanog* and *Klf4* (Figure 5F–I). EGI-1-derived spheres expressed higher levels of *CD24*, *CD44*, *ALDH1A1*, *EpCAM* and *Klf4* (Supplementary Materials Figure S2A–E). These findings indicate that the human CCA stem-like cells can be selectively enriched with stem cell serum-free medium.

### 3.6. Enhanced Expression of Immune Modulators in CCA Derived Stem-Like Cells

**Figure 6:**
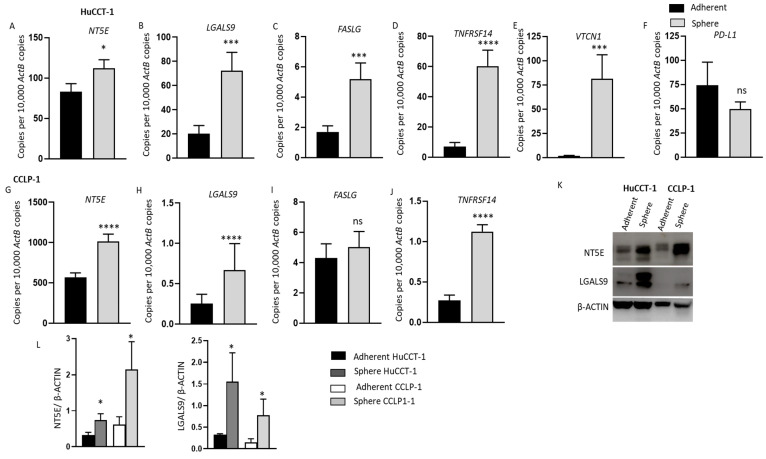
HuCCT-1 and CCLP-1 CSCs have elevated expression of immune modulators. (**A**–**F**) qRT-PCR analysis showed upregulation of immune modulators *NT5E*, *LGALS9*, *FASLG*, *TNFRSF14* and *VTCN1* in HuCCT-1 spheres compared with the parental adherent HuCCT-1 cells. (**G**–**J**) Increased expression of immune modulators *NT5E*, *LGALS9*, *FASLG* and *TNFRSF14* was detected in mRNA from CCLP-1 spheres versus the parental adherent cells. (**K**) Western blots demonstrated the upregulation of NT5E and LGALS9 in both HuCCT-1 and CCLP-1 spheres versus the adherent parental cells. (**L**) Graphs represent quantification of Western blot analyses. Values are mean ± SD of three experiments in triplicate (* *p* < 0.05, *** *p* < 0.005, **** *p* < 0.001, ns: not significant). *ActB: β-Actin*.

The expression of immune checkpoint regulators linked with poor outcome in patients with CCA was evaluated in CCA-derived CSCs. We compared the expression of immune checkpoints in spheres and adherent parental CCA cell lines using qRT-PCR. In HuCCT-1-derived spheres, we noted markedly increased expression of *NT5E*, *LGALS9*, *FASLG*, *TNFRSF14* and *VTCN1* compared with adherent cells (Figure 6A–E). However, the spheres showed lower *PD-L1* expression levels compared with adherent cells (Figure 6F). Similarly, elevated expression of *NT5E*, *LGALS9*, *FASLG* and *TNFRSF14* was detected in CCLP-1-derived spheres compared with parental adherent cells (Figure 6G–J). CCLP-1 cells did not express *PD-L1* and *VTCN1*. *IDO1*, *HAVCR2* and *CD80* expression was undetectable in all cell lines. Western blot assay showed increased NT5E and LGALS9 protein expression in CCA-derived CSCs from HuCCT-1 and CCLP-1 compared with adherent cells (Figure 6K,L). EGI-1-derived spheres showed elevated expression of *NT5E* (Supplementary Materials Figure S2F). No significant difference in expression of other immune checkpoint molecules was noted in EGI-1-derived CSCs and adherent cells (Supplementary Materials Figure S2G–H). Given the high expression of immune checkpoints in CCA-derived CSCs, ICI-based immunotherapy may be used to target the CSC population in CCA to obtain effective and durable treatment outcomes.

### 3.7. Induction of Immune Checkpoint Modulator Expression during TGF-β1- and TNF-α-Mediated EMT in Human CCA Cells

**Figure 7:**
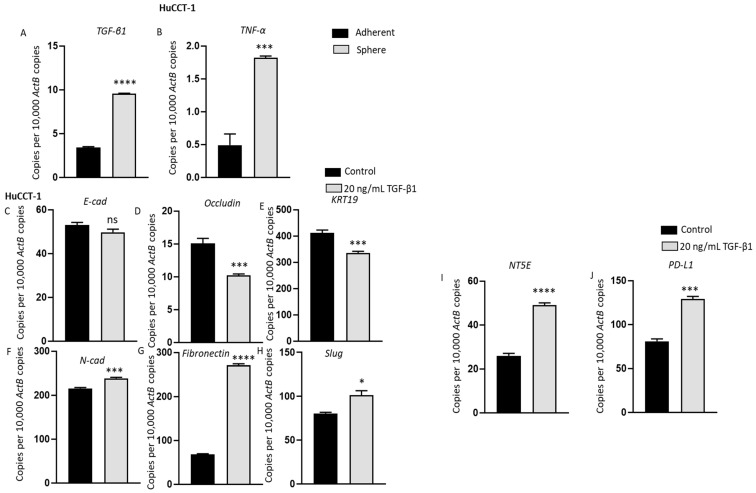
TGF-β1-mediated EMT induced upregulation of immune modulators in HuCCT-1 cells. qRT-PCR analysis revealed the upregulation in the expression of (**A**) *TGF-β* and (**B**) *TNF-α* in HuCCT-1 spheres compared with parent adherent cells. (**C**–**H**) qRT-PCR showed epithelial markers E-Cad, Occludin and KRT19 were decreased, and mesenchymal markers N-cad, Fibronectin and Slug were increased after 72 h of TGF-β1 treatment in HuCCT-1 cells. (**I**–**J**) qRT-PCR demonstrated elevated expression of immune modulators NT5E and PD-L1 upon 72 h of TGF-β1 treatment in HuCCT-1 cells. Values are mean ± SD of three experiments in triplicate (* *p* < 0.05, *** *p* < 0.005, **** *p* < 0.001, ns: not significant). *ActB:* *β-Actin*.

**Figure 8:**
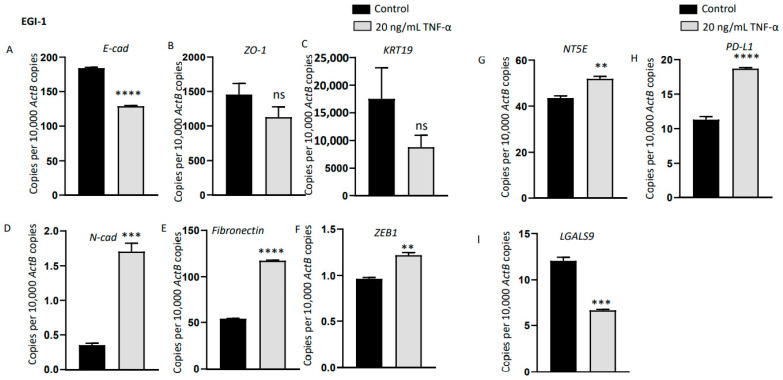
TNF-α-mediated EMT induced upregulation of immune modulators in CCLP-1 cells. (**A**–**F**) qRT-PCR showed reduction in the expression of epithelial markers *E-Cad*, *ZO-1* and *KRT19*, and elevation in the expression of mesenchymal markers *N-cad*, *Fibronectin* and *ZEB1* after 72 h of TNF-α treatment in CCLP-1 cells. (**G**–**I**) qRT-PCR demonstrated elevated expression of immune modulators *NT5E* and *PD-L1* and decreased level of *LGALS9* upon 72 h of TNF-α treatment in CCLP-1 cells. Values are mean ± SD of three experiments in triplicate (** *p* < 0.01, *** *p* < 0.005, **** *p* < 0.001, ns: not significant). *ActB: β-Actin*.

Emerging research has demonstrated a direct association between EMT and acquisition of stem-cell-like features [28,33]. We propose that the elevated expression of immune checkpoints in CSCs is regulated by EMT via TGF-β1 and/or TNF-α. EMT and CSC in turn enhance the invasion, metastasis and recurrence and may contribute to poor prognosis of CCA patients. Therefore, we investigated whether EMT inducers are expressed by CCA stem cells and if EMT can modulate immune checkpoint expression. For the subsequent study, we used cell lines that can undergo EMT upon induction with cytokines. Thus, we chose to study HuCCT-1 and EGI-1 that have an epithelial phenotype and excluded CCLP-1 cells with a mesenchymal phenotype. HuCCT-1 spheres showed upregulation in the expression of potent EMT drivers TGF-β and TNF-α compared with parent adherent cells (Figure 7A,B). Previously, EMT inducers have been shown to induce the expression of PD-L1 and other ICs in HCC [29]. In order to evaluate whether EMT is closely associated with modulation of ICs in CCA, HuCCT-1 cells were treated with 20ng/mL of TGF-β1. After 3 days, HuCCT-1 cells underwent EMT as evidenced by the decrease in epithelial markers *E-Cad*, *Occludin* and *KRT19* and elevation in mesenchymal markers *N-cad*, *Fibronectin* and *Slug* (Figure 7C–H). HuCCT-1 did not respond to TNF-α stimulation. Elevated expressions of ICs, *NT5E* and *PD-L1* were observed during TGF-β1-induced EMT in HuCCT-1 cells (Figure 7I,L). EGI-1 cells, on the other hand, were more responsive to TNF-α-driven EMT than TGF-β1. After stimulation of EGI-1 cells with 20 ng/mL TNF-α for 3 days, EGI-1 cells underwent EMT with a reduction in the expression of epithelial markers *E-Cad*, *ZO-1* and *KRT19* and upregulation in the expression of mesenchymal markers *N-cad*, *Fibronectin* and *Zeb1* (Figure 8A–F). TNF-α stimulation also induced *NT5E* and *PD-L1* expression (Figure 8G,H). These findings suggest that the expression of ICs in aggressive phenotypes such as cells undergoing EMT and stemness are closely linked.

### 3.8. Coordinate Expression of Immune Modulatory Genes, EMT Markers and Stemness Marker in CCA Patients

**Figure 9:**
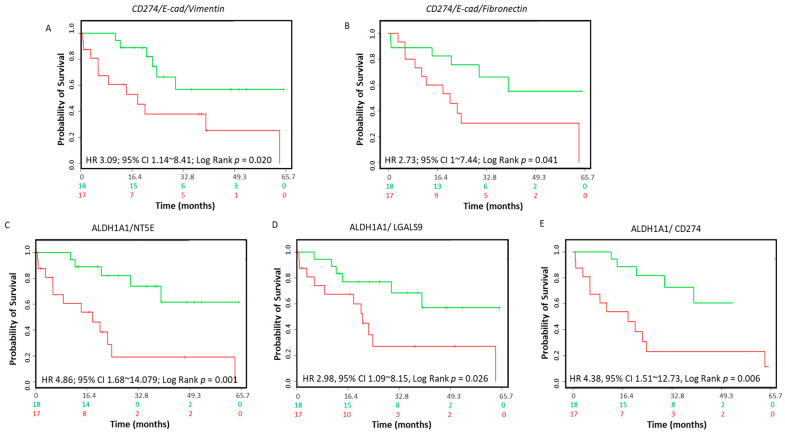
Association between immune modulators, EMT markers, CSC markers and survival in CCA patients. The Kaplan–Meier survival curves in SurvExpress CCA patients for the gene expression of (**A**) *CD274*/*E-Cad*/*Vimentin*, (**B**) *CD274*/*E-Cad*/*Fibronectin*, (**C**) *ALDH1A1*/*NT5E*, (**D**) *ALDH1A1*/*LGALS9*, (**E**) *ALDH1A1*/*CD274* in CCA patients. Low-risk groups are indicated in green and high-risk groups are indicated in red. The x-axis presents the study time in months. HR, CI and *p*-values are shown in the insert.

Elevated expression of PD-L1 has been recently reported in mesenchymal cells within CCA tumors [22]. We examined the association between PD-L1 with an EMT phenotype in a CCA patient cohort. Although expression of *PD-L1* alone was not significantly associated with poor prognosis in the CCA patient dataset, coordinate downregulation of E-Cad (*CDH1)* expression and upregulation of *VIM* expression revealed overall worse survival (HR: 3.09, CI: 1.14~8.41, log-rank equal curves *p* = 0.020) in combination with *PD-L1* (Figure 9A). Coordinate expression of *PD-L1* with *E-Cad* and *Fibronectin* also showed poor overall survival in these patients (HR: 2.73, CI: 1~7.44, log-rank equal curves *p* = 0.041) (Figure 9B). We next evaluated the relationship between immune checkpoint genes and stemness genes in CCA patients. Expression of stemness marker *ALDH1A1* showed poor overall survival when combined with *NT5E* (HR: 4.86, CI: 1.68~14.079, log-rank equal curves *p* = 0.001), *LASGALS9* (HR: 2.98, CI: 1.09~8.15, log-rank equal curves *p* = 0.026) or *PD-L1* (HR: 4.38, CI: 1.51~12.73, log-rank equal curves *p* = 0.006) (Figure 9C–E). Coordinate expression of other stemness genes including *CD13*, *CD24*, *CD133*, *EpCAM*, *OCT4*, *SOX2*, *Nanog* and *KLF4* with immune checkpoints evaluated in this study did not show an association with overall survival in CCA patients (Supplementary Materials Table S2). There was no significant association of overall survival of CCA patients showing coordinate expressions of CSC markers and immune checkpoint genes *FASLG*, *TNRSF14*, *VTCN1* and *PD-L1* (Supplementary Materials Table S2). These findings reveal that elevated *PD-L1* expression in CCA patients is closely linked with EMT status, and high co-expression of *PD-L1*, *NT5E* or *LGALS9* with stemness marker *ALDH1A1* is related to poor prognosis in CCA patients.

## 4. Discussion

In the present pilot study, the expression of immune modulators *IDO1*, *NT5E* and *FASLG* was related to poor prognosis in CCA patients. Furthermore, a combination of various ICs with putative immune modulators PD-1, PD-L1 and CTLA-4 was associated with poor CCA patient prognosis. Moreover, EMT and CSC were closely associated with the modulation of immune checkpoint molecules in CCA. PD-L1 and NT5E expression was closely associated with EMT, while coordinate expression of NT5E and LSGAL9 with CSC marker ALDH1A1 was linked with poor overall survival in CCA patients.

Indoleamine 2,3-dioxygenase 1 (IDO1) is an intracellular heme-containing enzyme that contributes to the immune escape of tumors [34]. Our observation of the association of high IDO1 with poor overall survival in CCA patients is consistent with observations in colorectal, non-small-cell lung and prostate cancers [35]. In contrast, high IDO1 expression levels in HCC patients have been correlated with better survival outcomes, indicating that IDO1 may not have immunosuppressive functions in this cancer [36,37]. Elevated IDO1 expression in cancers has been correlated with single-agent ICI therapy resistance [38]. Our findings suggest that combining IDO1 inhibitors with other ICIs may represent a promising strategy to expand CCA patient populations for immunotherapies. FASLG, a transmembrane protein of the tumor necrosis factor superfamily triggers apoptosis of T-cells [39]. Ecto-5′-nucleotidase (CD73 or NT5E) is a glycophosphatidylinositol-anchored receptor enzyme that blocks activation of T-cell when adenosine binds to its receptor [40]. A study found that CCA cell lines that expressed FASLG, induced cell death when cocultured with T-cells, indicative of the immune evasive function of FASLG/FAS axis in CCA [39]. We and others have previously found that NT5E expression in cancers was associated with poor prognosis [10,41]. We found that IDO1 expression was associated with clinical parameters, such as a presence of risk factors for HCC and tumor stage II and III, while the expression of FASLG in CCA patients was associated with the lymph node stage. IDO1, FASLG and NT5E showed no association with clinical parameters including vascular invasion, gender and tumor stage T0–T3.

This pilot study revealed that IDO1, FASLG, CD80, HAVCR2, CD73, CTLA-4, LGALS9, VTCN1 and TNFRSF14 in combination with PD-L1 is linked with poor outcome in CCA patients. The Cluster of differentiation 80 (CD80) regulates T-cell activation by binding to CTLA4. A study on biliary tract cancers including CCA reported that strong CD80 expression in tumor tissue was closely associated with resistance to adjuvant chemotherapy [42]. The T-cell immunoglobulin and mucin-domain 3 (TIM3 or HAVCR2) receptor limits T-cell responses by interacting with its ligand Galectin-9 (LGALS9 or Gal-9) [43]. In animal models, combining anti-HAVCR2 and anti-PD-1 has shown to suppress tumor growth [44].

V-set domain-containing T-cell activation inhibitor 1, VTCN1, (also named B7-H4, B7S1 or B7x) belongs to the B7 family and regulates T-cell-mediated antitumor responses. Studies in CCA patients showed high levels of VTCN1 expression were significantly related to poor prognosis [45,46]. LGALS9 is a tandem-repeat-type galectin that promotes antitumor immune responses by exerting antiproliferative effects on CAA cells [47]. T-cell immunoglobulin and ITIM domain (TIGIT) is an inhibitory immune checkpoint of the poliovirus receptor (PVR)/desmin family [48]. Inhibition of TIGIT alone or with PD-1 has shown to restore tumor-suppressive effects [49]. The tumor stroma of CCA patients showed infiltration of lymphocytes expressing ICs, including PD1 and TIGIT [50]. The function of tumor necrosis factor receptor superfamily member 14 (TNFRSF14) is not known in CCA.

We and others have noted PD-L1 expression was closely linked with EMT status [22]. This is the first study to examine the relationship between TGF-β1- and TNF-α-induced EMT and upregulation of PD-L1 and NT5E expression. Furthermore, we and others have reported that PD-L1 expression negatively impacts CCA patient prognosis [51]. In contrast, other studies show that CCA patients with low PD-L1 expression had a poorer prognosis [24,52].

A study showed that CCA-derived, CD133-positive CSCs displayed high levels of TGF-β1 and activation of the TGF-β1–pSmad2–EMT axis [53]. Similarly, we found CCA-derived CSCs showed a high expression of TGF-β1 along with TNF-α. A study reported that the PD-L1 low cell fraction isolated from HuCCT-1 cells was enriched with CSC-related characteristics compared with the PD-L1 high cell fraction [24]. This observation is consistent with our results demonstrating the downregulation of PD-L1 in CSCs enriched by HuCCT-1 cells. This study is the first to examine the relationship between CSC phenotype and other novel ICs including NT5E, LGALS9, TNFRSF14, FASLG and VTCN1. Our data suggest that CCA patient tumors with mesenchymal and CSC phenotypes might be targeted using immune checkpoint blockades.

This study is limited by a lack of CCA patient samples who have undergone treatment with immune checkpoint therapies. This pilot study, comprising a small number of CCA patients, may not provide adequate information and conclusions. Thus, further validation of the potential of immune checkpoint regulators as prognostic markers in larger cohorts of CCA patients will be more informative. Another limitation of this study is the lack of available clinical data for the SurvExpress CCA patients. Furthermore, the prognosis of CCA may be affected by the location of the tumor. In this study, we were unable to evaluate the independent association between distal, perihilar or intrahepatic CCA with the expression of immune checkpoints and patient prognosis. We were unable to assess the association between immune checkpoint expression and cancer-specific mortality. Further studies are needed to evaluate the correlation between immune checkpoint expression with parameters such as tumor location and cancer-specific mortality. While this study focused on the expression ICs in CCA tumor cells, comprehensive investigation is needed to validate the role of each individual IC in in vitro and in vivo CCA models. Studies particularly focusing on molecular mechanisms, functional assays, including motility and drug testing, and evaluation of ICs in animal models will be required to gain a better understanding of their function in CCA tissues. Further studies are needed to assess the expression of these molecules on tumor-infiltrating T-cells, which will also be helpful in predicting ICI responses. Additionally, studies on the co-expression of these immune checkpoints in CCA, as well as on how they influence or act with each other, will need to be assessed to enable effective and durable treatment.

## 5. Conclusions

CCA has a dismal prognosis with very limited therapeutic options. Accordingly, novel treatment modalities that are both effective and associated with durable responses are needed for the treatment of CCA. Immunotherapy in the form of ICIs is anticipated to be used as an effective treatment modality for CCA. Immune checkpoint molecules IDO1, FASLG, CD80, HAVCR2, CD73, CTLA-4, LGALS9, VTCN1, TNFRSF14 and PD-L1 may be useful as potential biomarkers for the treatment and prognosis of CCA patients. Additionally, ICI-based immunotherapy may be used to target EMT and CSC populations in CCA to achieve lasting and durable treatment outcomes. This study is the first attempt to examine the association between immune checkpoint modulator expression, CSCs and EMT status in CCA, providing a better understanding of the molecular events contributing to prognosis prediction. Further investigations of the underlying above-detailed mechanisms leading to overexpression of immune checkpoints in CCA microenvironment will provide better strategies for ICI therapy.

## Figures and Tables

**Table 1:** List of qRT-PCR primers.

Primers	Forward Sequence (5′–3′)	Reverse Sequence (5′–3′)
*ActB*	CCAACCGCGAGAAGATGA	CCAGAGGCGTACAGGGATAG
*CD13*	CAGTGACACGACGATTCTCC	CCTGTTTCCTCGTTGTCCTT
*CD24*	CACGCAGATTTATTCCAGTGAAAC	GACCACGAAGAGACTGGCTGTT
*CD44*	CACGTGGAATACACCTGCAA	GACAAGTTTTGGTGGCACG
*CD90*	AGGACGAGGGCACCTACAC	GCCCTCACACTTGACCAGTT
*CD133*	GCTTCAGGAGTTTCATGTTGG	GGGGAATGCCTACATCTGG
*ALDH1A1*	CGGGAAAAGCAATCTGAAGAGGG	GATGCGGCTATACAACACTGGC
*EpCAM*	CCCATCTCCTTTATCTCAGCC	CTGAATTCTCAATGCAGGGTC
*OCT4*	TTGTGCCAGGGTTTTTGG	ACTTCACCTTCCCTCCAACC
*SOX2*	ATGGGTTCGGTGGTCAAGT	GGAGGAAGAGGTAACCACAGG
*NANOG*	CTCCAACATCCTGAACCTCAGC	CGTCACACCATTGCTATTCTTCG
*KLF4*	CATCTCAAGGCACACCTGCGAA	TCGGTCGCATTTTTGGCACTGG
*ZO1*	GTCCAGAATCTCGGAAAAGTGCC	CTTTCAGCGCACCATACCAACC
*KRT19*	GGTCAGTGTGGAGGTGGATT	TCAGTAACTCGGACCTGCT
*E-Cad*	AGGCCAAGCAGCAGTACATT	ATTCACATCCAGCACATCCA
*N-cad*	TCCTTGCTTCTGACAATGGA	TTCGCAAGTCTCTGCCTCTT
*Occludin*	TAGTCAGATGGGGGTGAAGG	CATTTATGATGAGCAGCCCC
*ZEB1*	GGCATACACCTACTCAACTACGG	TGGGCGGTGTAGAATCAGAGTC
*Slug*	TGGTTGCTTCAAGGACACAT	GTTGCAGTGAGGGCAAGAA
*Fibronectin*	CAGTGGGAGACCTCGAGAAG	TCCCTCGGAACATCAGAAAC
*LGALS9*	ACACCCAGATCGACAACTCCTG	CAAACAGGTGCTGACCATCCAC
*TNFRSF14*	TTCTCTCAGGGAGCCTCGTCAT	CTCACCTTCTGCCTCCTGTCTT
*FASLG*	CCTTGGTAGGATTGGGCCTG	TCTGGCTGGTAGACTCTCGG
*TGF-β1*	TACCTGAACCCGTGTTGCTCTC	GTTGCTGAGGTATCGCCAGGAA
*TNF-α*	CCCAGGGACCTCTCTCTAATC	TCTCAGCTCCACGCCATT
*PD-L1*	GCTGCACTAATTGTCTATTGGGA	AATTCGCTTGTAGTCGGCACC
*NT5E*	TTGGAAATTTGGCCTCTTTG	ACTTCATGAACGCCCTGC
*VTCN1*	TCTGGGCATCCCAAGTTGAC	TCCGCCTTTTGATCTCCGATT

**Table 2:** List of antibodies.

Antibodies	Cat. No.	Manufacturer	Antibody Category	Dilution
NT5E	ab175396	Abcam	Primary	1:6000
LGALS9	ab227046	Abcam	Primary	1:1000
β-Actin	4967s	Cell Signaling	Primary	1:4000
Goat anti-mouse HRP	62-6520	Invitrogen	Secondary	1:50,000
Goat anti-rabbit HRP	65-6120	Invitrogen	Secondary	1:50,000

**Table 3:** Clinical characteristics of 51 CCA patients.

CCA Patient ID	Diagnosed Age	Sex	Ablation Embolization Tx Adjuvant	Surgical Margin Resection Status	Adjuvant Postoperative Pharmaceutical Therapy Administered Indicator	American Joint Committee on Cancer Tumor Stage Code	American Joint Committee on Cancer Metastasis Stage Code
TCGA-3X-AAV9	72	Male	NO	R0	YES	T1	M0
TCGA-5A-A8ZF	NA	NA	NA	NA	NA	NA	NA
TCGA-5A-A8ZG	NA	NA	NA	NA	NA	NA	NA
TCGA-W7-A93N	NA	NA	NA	NA	NA	NA	NA
TCGA-W7-A930	NA	NA	NA	NA	NA	NA	NA
TCGA-W7-A93P	NA	NA	NA	NA	NA	NA	NA
TCGA-ZK-AAYZ	NA	NA	NA	NA	NA	NA	NA
TCGA-3X-AAVA	50	Male	NO	R0	YES	T1	M0
TCGA-3X-AAVB	70	Female	NO	R0	NO	T2b	M1
TCGA-3X-AAVC	72	Female	NA	R0	NA	T3	M0
TCGA-3X-AAVE	60	Female	NO	R0	NO	T1	M0
TCGA-4G-AAZF	74	Male	NO	R0	NO	T2	MX
TCGA-4G-AAZG	75	Female	NO	R0	NO	T3	MX
TCGA-4G-AAZ0	71	Female	NO	R1	YES	T2a	M0
TCGA-4G-AAZR	74	Male	NO	R1	NO	T2a	MX
TCGA-4G-AAZT	62	Male	NO	R0	NO	T1	M0
TCGA-W5-AA2G	62	Female	NO	R0	NO	T1	M0
TCGA-W5-AA2H	70	Female	NO	R0	YES	T3	M0
TCGA-W5-AA2I	66	Male	NO	R0	NO	T1	M0
TCGA-W5-AA2J	66	Female	NO	R0	NO	T4	M0
TCGA-W5-AA2K	75	Female	NO	R0	NO	T1	M0
TCGA-W5-AA2M	49	Male	NO	R0	NO	T3	M1
TCGA-W5-AA2O	57	Male	NO	R0	NO	T1	M0
TCGA-W5-AA2Q	68	Male	NO	R0	NO	T2b	M0
TCGA-W5-AA2R	77	Female	NO	R0	NO	T1	M0
TCGA-W5-AA2T	64	Female	NO	R0	YES	T2	M0
TCGA-W5-AA2U	78	Female	NO	R0	NO	T1	M0
TCGA-W5-AA2W	31	Female	NO	R0	NO	T2a	M0
TCGA-W5-AA2X	67	Male	NO	RX	NO	T2b	M1
TCGA-W5-AA2Z	29	Female	NO	R1	YES	T2	M0
TCGA-W5-AA30	82	Male	NO	R0	NO	T1	M0
TCGA-W5-AA31	71	Male	NO	R0	NO	T1	M0
TCGA-W5-AA33	60	Male	NO	R0	NO	T1	M0
TCGA-W5-AA34	75	Female	NO	R0	NO	T1	M0
TCGA-W5-AA36	51	Female	NO	R0	YES	T3	M1
TCGA-W5-AA38	55	Female	NO	R0	NO	T1	M0
TCGA-W5-AA39	81	Male	NO	RX	NO	T2	M0
TCGA-W6-AA0S	46	Female	NO	R0	YES	T1	Mx
TCGA-W6-AA0T	62	Female	NO	R0	YES	T3	M0
TCGA-WD-A7RX	71	Female	NO	RX	NO	T2b	MX
TCGA-YR-A95A	52	Male	NO	R1	NO	T2	M1
TCGA-ZD-A8I3	73	Female	NA	R0	NA	T2	MO
TCGA-ZH-A8Y1	74	Female	NO	R1	NO	T3	MO
TCGA-ZH-A8Y2	59	Female	NO	R0	NO	T1	MO
TCGA-ZH-A8Y3	61	Female	NO	R1	YES	T2a	MO
TCGA-ZH-A8Y4	58	Male	NO	R1	YES	T1	MO
TCGA-ZH-A8Y5	69	Male	NO	R0	YES	T3	M1
TCGA-ZH-A8Y6	41	Female	NA	R0	NA	T1	MO
TCGA-ZH-A8Y7	59	Male	NO	R1	NO	T3	M1
TCGA-ZH-A8Y8	73	Male	NO	R0	NO	T1	MO
TCGA-ZU-A8S4	52	Male	NO	R0	YES	T1	MX

NA: Clinical data not available for the patient.

**Table 4:** SurvExpress-based overall survival of 35 CCA patients.

Immune Modulatory Gene	Risk Groups Hazard Ratio	Confidence Interval	Log-Rank Equal Curves (*p*-Value)
*FASLG*	1.27	0.17–9.69	0.817
*LAG3*	0.84	0.32–2.18	0.720
*HAVCR2*	1.87	0.71–14.96	0.198
*VSIR*	1.69	0.61–4.68	0.309
*VTCN1*	0.87	0.33–2.27	0.776
*TNFRSF9*	81853258	0–∞	0.132
*TNFRSF14*	1.39	0.53–3.66	0.503
*TIGIT*	1.37	0.5–3.71	0.536
*CD276*	0.67	0.26–1.75	0.414
*CD27*	1.15	0.42–3.11	0.785
*PDCD1LG2*	1.53	0.55–4.21	0.409
*NT5E*	0.56	0.21–1.5	0.247
*CD80*	3.41	0.45–25.04	0.208
*TNFRSF18*	1.92	0.73–5.08	0.178
*BTLA*	1.36	0.31–5.97	0.686
*CD28*	2.3	0.8–6.64	0.112

**Table 5:** cBioPortal-based CCA patient overall survival and progression-free survival.

Immune Modulatory Gene	Overall Survival Log-Rank Test (*p*-Value)	Progression-Free Survival Log-Rank Test (*p*-Value)
*FASLG*	1.370 × 10^−3^	3.843 × 10^−3^
*LGALS9*	0.286	0.759
*LAG3*	0.464	0.898
*HAVCR2*	0.095	0.225
*VSIR*	0.515	0.290
*VTCN1*	0.361	0.900
*IDO1*	0.450	0.242
*TNFRSF9*	NA	NA
*TNFRSF14*	NA	NA
*TIGIT*	0.515	0.290
*CD276*	0.514	0.290
*CD27*	0.515	0.290
*CD274*	0.054	0.109
*PDCD1LG2*	0.464	0.898
*NT5E*	2.826 × 10^−3^	4.072 × 10^−3^
*CD80*	0.515	0.290
*TNFRSF18*	NA	NA
*BTLA*	0.515	0.290
*CD28*	0.515	0.290

NA: No patient data available with alteration in this gene.

**Table 6:** Relationship between expression of IDO1, FASLG and NT5E and clinical parameters in CCA patients.

Clinical Attribute	*p* = 1.874 × 10^−3^		*p* = 0.937		*p* = 0.969	
Neoplasm disease stage American Joint Committee on Cancer Code	Altered IDO1	Unaltered	Altered FASLG	Unaltered	Altered NT5E	Unaltered
Stage I	0%	55.88%	20%	58.06%	100%	51.43%
Stage II	50%	23.53%	60%	19.35%	0%	25.71%
Stage III	50%	0%	0%	3.23%	0%	2.86%
Stage IV	0%	5.88%	0%	6.45%	0%	5.71%
Stage IVA	0%	5.88%	20%	3.23%	0%	5.71%
Stage IVB	0%	8.82%	0%	9.68%	0%	8.57%
	*p* = 0.739		*p* = 0.018		*p* = 3.492 × 10^−3^	
Metastasis stage American Joint Committee on Cancer Code	Altered IDO1	Unaltered	Altered FASLG	Unaltered	Altered NT5E	Unaltered
M0	100%	76.47%	60%	80.65%	0%	80%
M1	0%	14.71%	0%	16.13%	0%	14.29%
MX (No information available)	0%	8.82%	40%	3.23%	100%	5.71%
	*p* = 0.665		*p* = 0.041		*p* = 0.041	
Neoplasm disease lymph node stage American Joint Committee on Cancer Code	Altered IDO1	Unaltered	Altered FASLG	Unaltered	Altered NT5E	Unaltered
N0	0%	70.59%	40%	77.42%	0%	74.29%
N1	74.29%	14.71%	20%	12.9%	0%	14.29%
NX (No information available)	14.29%	14.71%	40%	9.68%	100%	11.43%
	*p* = 0.301		*p* = 0.937		*p* = 0.922	
Tumor stage American Joint Committee on Cancer Code	Altered IDO1	Unaltered	Altered FASLG	Unaltered	Altered NT5E	Unaltered
T1	0%	55.88%	20%	58.06%	100%	51.43%
T2	50%	14.71%	0%	19.35%	0%	17.14%
T2a	0%	5.88%	40%	0%	0%	5.71%
T2b	0%	11.76%	40%	6.45%	0%	11.43%
T3	50%	11.76%	0%	16.13%	0%	14.29%
	*p* = 0.049		*p* = 1.046 × 10^−3^		*p* = 3.565 × 10^−3^	
Neoadjuvant therapy type administered prior to resection	Altered IDO1	Unaltered	Altered FASLG	Unaltered	Altered NT5E	Unaltered
YES	0%	2.94%	0%	2.86%	0%	2.86%
NO	100%	97.06%	100%	97.14%	100%	97.14%
	*p* = 0.012		*p* = 1.00		*p* = 0.998	
History hepatocellular risk factor	Altered IDO1	Unaltered	Altered FASLG	Unaltered	Altered NT5E	Unaltered
Hepatitis B and smoking	50%	0%	60%	6.67%	0%	26.47%
No history of primary risk factors	50%	58.82%	20%	63.33%	100%	55.88%
Other risk factors (cirrhosis/diabetes mellitus/NAFLD/ smoking/ulcerative colitis)	0%	41.1%	20%	30%	0%	17.65%
	*p* = 0.569		*p* = 0.910		*p* = 0.930	
Gender	Altered IDO1	Unaltered	Altered FASLG	Unaltered	AlteredNT5E	Unaltered
Male	50%	44.12%	40%	45.16%	100%	44.12%
Female	50%	55.88%	60%	54.84%	0%	55.88%

## Data Availability

The data presented in this study are available in this article and supplementary material.

## Supplementary Materials

The following are available online at https://www.mdpi.com/article/10.3390/jcm10102191/s1, Figure S1: Link between ICs in combination and survival in CCA patients. The Kaplan–Meier survival curves generated with the SurvExpress CCA patient dataset for the gene expression of (A) *PD-1*/*TIGIT*, (B) *PD-1*/*TNFRSF14*, (C) *PD-1*/*CTLA4*, (D) *PD-1*/*FASLG*, (E) *CTLA4*/*IDO1* and (F) *CTLA4*/*CD274*. Green indicates low-risk group. Red indicates high-risk group. The x-axis shows the study time in months. HR, CI and P values are shown in the insert. Figure S2: Enrichment of CSCs with anchorage-independent three-dimensional spheroid culture in EGI-1 cells. (A) Monolayer culture and 3-D culture of EGI-1 cells (scale bar = 200 µm). (B-F) Increased expression of embryonic stemness and cell surface CSC markers was detected in EGI-1 spheres compared with adherent monolayer EGI-1 cells. (G-I) qRT-PCR detected higher expression of *NT5E*. Values are mean ± SD of three experiments in triplicate (* *p* < 0.05, ** *p* < 0.01, **** *p* < 0.001, ns: not significant). *ActB*: β-Actin. Table S1: SurvExpress-based overall survival of 35 CCA patients showing co-ordinate expression of *PD-L1* and other ICs. Table S2: SurvExpress-based overall survival of 35 CCA patients showing co-ordinate expression of ICIs and CSC markers.
