# Supplementary material for: Prognostic Role of Immune Checkpoint Regulators in Cholangiocarcinoma: A Pilot Study
Source: J Clin Med. 2021 May 19;10(10):2191. doi: 10.3390/jcm10102191 (PMC8159105; doi:10.3390/jcm10102191)
Supplement: Supplementary file 1 [file jcm-10-02191-s001.zip › jcm-1175549-supplementary.pdf]

Journal of
Clinical Medicine

MDPI

Article

Supplementary Material: Prognostic Role of Immune Checkpoint Regulators in Cholangiocarcinoma: A Pilot Study

Lu Cao, Prashanth Prithviraj, Ritu Shrestha, Revati Sharma, Matthew Anaka, Kim R. Bridle, George Kannourakis, Darrell H.G. Crawford and Aparna Jayachandran

Table S1. SurvExpress-based overall survival of 35 CCA patients showing co-ordinate expression of *PD-L1* and other ICs.

Immune Modulatory Gene	Risk Groups Hazard Ratio	Confidence Interval	Log-Rank Equal Curves (*p*-Value)
LAG3	2.11	0.78–5.74	0.133
VSIR	2.68	0.93–7.74	0.067
TNFRSF9	NA	NA	NA
TNFRSF18	2.38	0.86–6.58	0.094
TIGIT	2.75	0.95–7.95	0.061
CD276	1.5	0.58–3.89	0.406
CD27	1.83	0.66–5.05	0.243
PDCD1LG2	2.36	0.83–6.7	0.108
BTLA	NA	NA	NA
CD28	1.95	0.72–5.28	

NA: No patient data available with alteration in this gene.

Table S2. SurvExpress-based overall survival of 35 CCA patients showing co-ordinate expression of ICIs and CSC markers.

Immune Modulatory Gene	Risk Groups Hazard Ratio	Confidence Interval	Log-Rank Equal Curves (*p*-Value)
NT5E			
CD13	0.56	0.21–1.5	0.240
CD24	0.99	0.38–2.58	0.981
CD133	0.9	0.34–2.41	0.835
EpCAM	0.85	0.32–2.27	0.743
OCT4	0.75	0.28–2.03	0.573
SOX2	0.8	0.3–2.13	0.650
NANOG	0.56	0.21–1.5	0.247
KLF4	1.27	0.48–3.34	0.633
LGALS9			
CD13	NA	NA	NA
CD24	1.51	0.56–4.06	0.415
CD133	NA	NA	NA
EpCAM	0.95	0.35–2.56	0.919
OCT4	1.37	0.53–3.57	0.516
SOX2	0.8	0.3–2.13	0.650
NANOG	1.1	0.41–12.94	0.851
KLF4	1.22	0.45–3.26	0.693
FALSG			
CD13	NA	NA	NA
CD24	1.01	0.39–2.63	0.977

CD133	0.68	0.26–1.77	0.423
EpCAM	1.16	0.45–3.02	0.756
OCT4	0.93	0.35–2.49	0.885
SOX2	1.33	0.17–10.28	0.781
NANOG	NA	NA	NA
KLF4	0.97	0.36–2.63	0.956
TNFRSF14			
CD13	NA	NA	NA
CD24	0.8	0.3–2.14	0.650
CD133	1.39	0.53–3.66	0.503
EpCAM	1.62	0.59–4.46	0.347
OCT4	1.04	0.4–2.7	0.937
SOX2	2.06	0.3–2.13	0.650
NANOG	NA	NA	NA
KLF4	1.98	0.68–5.74	0.199
VTCN1			
CD13	NA	NA	NA
CD24	0.81	0.31–2.12	0.672
CD133	0.87	0.33–2.27	0.776
EpCAM	NA	NA	NA
OCT4	1.12	0.42–2.99	0.817
SOX2	1.17	0.45–3.05	0.750
NANOG	0.87	0.33–2.27	0.776
KLF4	1.3	0.49–3.43	0.591
PD-L1			
CD13	NA	NA	NA
CD24	1.49	0.57–3.87	0.414
CD133	2.65	0.92–7.65	0.061
EpCAM	0.95	0.35–2.56	0.919
OCT4	2	0.72–5.55	0.172
SOX2	4.85	0.58–40.35	0.105
NANOG	1.1	0.41–12.94	0.851
KLF4	2.29	0.85–6.13	0.090

NA: No patient data available with alteration in this gene.

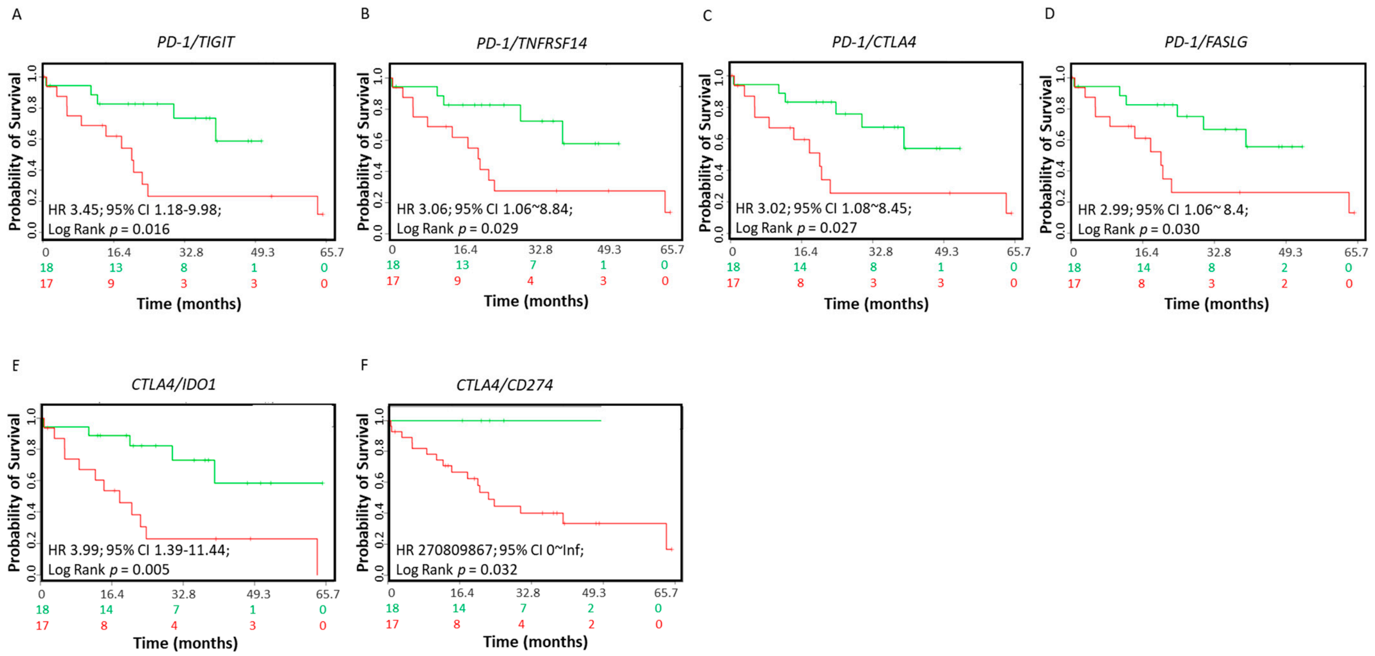

Figure S1. Link between ICs in combination and survival in CCA patients. The Kaplan–Meier survival curves generated with the SurvExpress CCA patient dataset for the gene expression of (**A**) *PD-1/TIGIT*, (**B**) *PD-1/TNFRSF14*, (**C**) *PD-1/CTLA4*, (**D**) *PD-1/FASLG*, (**E**) *CTLA4/IDO1* and (**F**) *CTLA4/CD274*. Green indicates low-risk group. Red indicates high-risk group. The x-axis shows the study time in months. HR, CI and *p* values are shown in the insert.

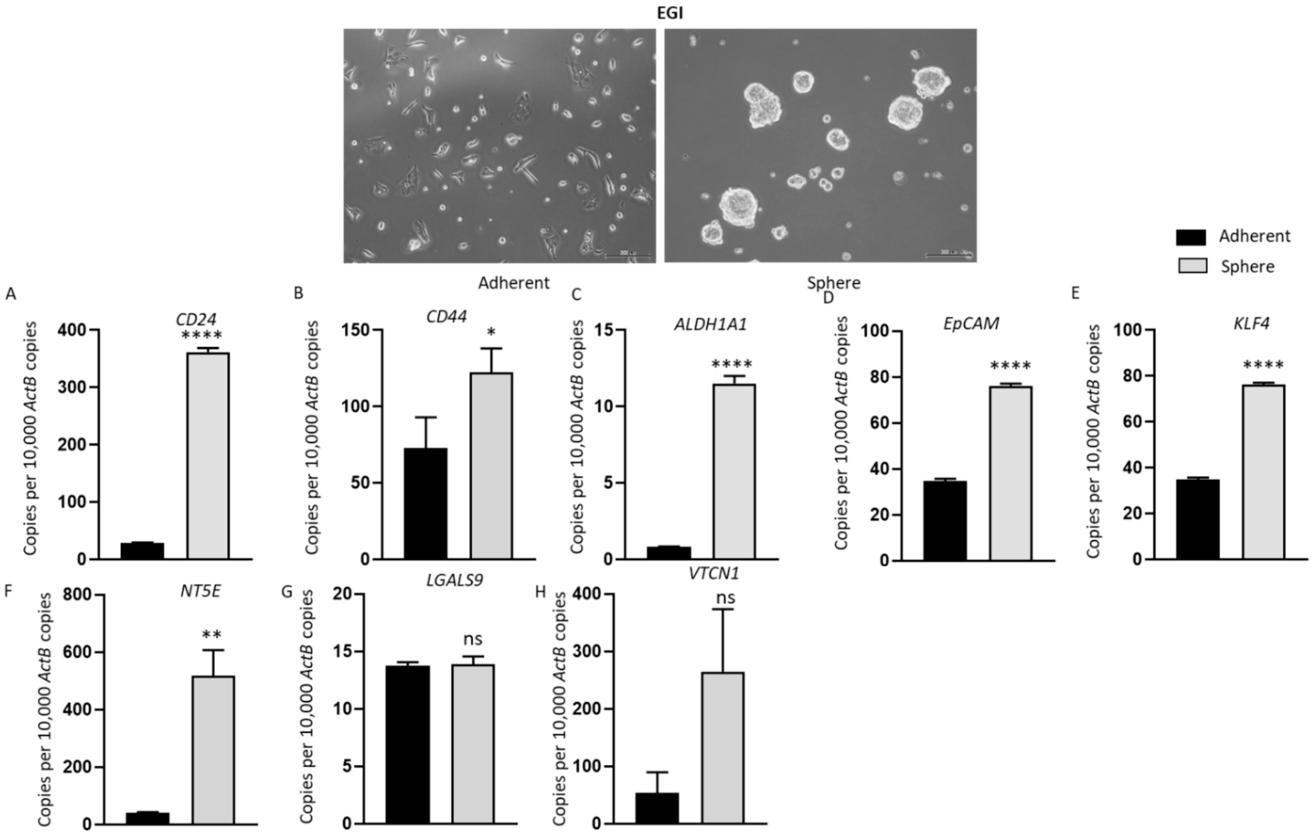

Figure S2. Enrichment of CSCs with anchorage-independent three-dimensional spheroid culture in EGI-1 cells. (**A**) Monolayer culture and 3-D culture of EGI-1 cells (scale bar = 200 μm). (**B**–**F**) Increased expression of embryonic stemness and cell surface CSC markers was detected in EGI-1 spheres compared with adherent monolayer EGI-1 cells. (**G**–**I**) qRT-PCR

detected higher expression of *NT5E*. Values are mean ± SD of three experiments in triplicate (* $p < 0.05$, ** $p < 0.01$, **** $p < 0.001$, ns: not significant). *ActB*: *β-Actin*.
